# Interactive Alignment and Lexical Triggering of Code-Switching in Bilingual Dialogue

**DOI:** 10.3389/fpsyg.2020.01747

**Published:** 2020-07-22

**Authors:** Gerrit Jan Kootstra, Ton Dijkstra, Janet G. van Hell

**Affiliations:** ^1^Centre for Language Studies, Radboud University, Nijmegen, Netherlands; ^2^Donders Centre for Cognition, Radboud University, Nijmegen, Netherlands; ^3^Department of Psychology, The Pennsylvania State University, University Park, PA, United States; ^4^Center for Language Science, The Pennsylvania State University, University Park, PA, United States

**Keywords:** code-switching, bilingual, interactive alignment, priming, cognates, dialogue

## Abstract

When bilingual speakers use two languages in the same utterance, this is called code-switching. Previous research indicates that bilinguals’ likelihood to code-switch is enhanced when the utterance to be produced (1) contains a word with a similar form across languages (lexical triggering) and (2) is preceded by a code-switched utterance, for example from a dialogue partner (interactive alignment/priming of code-switching). Both factors have mostly been tested on corpus data and have not yet been studied in combination. In two experiments, we therefore investigated the combined effects of interactive alignment and lexical triggering on code-switching. In Experiment 1, Dutch-English bilinguals described pictures to each other in a dialogue game where a confederate’s code-switching was manipulated. The participants were free to use either Dutch, English, or a combination of Dutch and English in describing the pictures, so they could voluntarily code-switch or not. The pictures contained a cognate [e.g., *roos* (rose)], a false friend [e.g., *rok* (skirt, false friend with *rock*)], or a control word [e.g., *jas* (coat)]. Participants code-switched more often when the confederate had just code-switched (indicating interactive alignment). They also code-switched more often when cognates were involved, but only when the confederate had just code-switched. This indicates that lexical triggering is driven by interactive alignment. False friends did not enhance the likelihood of code-switching. Experiment 2 used a similar dialogue game with participants from the same population but focused specifically on how to account for interactive alignment of code-switching. Rather than aligning on their dialogue partner’s pragmatic act of code-switching, bilinguals aligned on the language activation from the utterance produced by their dialogue partner. All in all, the results show how co-activation of languages at multiple levels of processing together influence bilinguals’ tendency to code-switch. The findings call for a perspective on bilingual language production in which cross-speaker and cross-language processes are combined.

## Introduction

A hallmark of bilingual language production is code-switching, which can be defined as the mixing and merging of two languages into one sentence. Code-switching can be observed in the speech of both low- and high-proficient bilinguals and across many language pairs (see Muysken, 2000; Gardner-Chloros, 2009, for overviews), and shows the remarkable flexibility and adaptability of how people use language. Being such a pervasive phenomenon of bilingual speech, code-switching has inspired bilingualism research in many disciplines, including linguistics (e.g., Poplack, 1980; Muysken, 2000; Myers-Scotton, 2002; Goldrick et al., 2016), sociolinguistics (e.g., Blom and Gumperz, 1972; Myers-Scotton, 1993; Auer, 1998; Tseng and Cashman, 2015; Torres Cacoullos and Travis, 2016), psycholinguistics (e.g., Kootstra et al., 2010, 2012; Hatzidaki et al., 2011; Green and Wei, 2014; Guzzardo Tamargo et al., 2016; Broersma et al., 2019), and neurocognition (e.g., Moreno et al., 2002; Litcofsky and Van Hell, 2017; Liu et al., 2018; Fernandez et al., 2019).

Code-switching is particularly interesting from a psycholinguistic perspective in that it is a natural phenomenon that overtly reflects language co-activation. Co-activation of languages refers to the well-established phenomenon that, during language production and comprehension, both a bilingual’s languages are active and influence language processing (see e.g., Kroll et al., 2006, 2012; Dijkstra, 2007; Hartsuiker and Pickering, 2008; Jiang, 2015; Van Gompel and Arai, 2018, for reviews). Code-switching is one of the most prominent natural discourse phenomena in which this co-activation of language elements is overtly reflected in real life, and as such, models of bilingual language production should be able to account for it. What is more, code-switching inherently involves multiple levels of processing: It involves socio-interactional considerations and is influenced by the properties of the words and linguistic structures in the code-switched utterance (Gardner-Chloros, 2009). Code-switching thus provides an ideal test bed to study the cognitive mechanisms of bilingual language production at multiple levels of processing.

A fundamental question within this psycholinguistic perspective is which factors at which levels of processing influence bilinguals’ tendency to code-switch. At the level of sentence production, one line of research has studied to what extent cross-language lexical overlap influences the likelihood to code-switch, as specified in the lexical triggering hypothesis of code-switching (e.g., Clyne, 1980; Broersma and De Bot, 2006; Broersma et al., 2019). Lexical triggering refers to the mechanism by which language-ambiguous words [e.g., cognates^1^, translations that overlap in phonology (and often also in orthography) across languages, like the English-Dutch “apple”-“appel”] facilitate, or trigger, a speaker to switch from one language to the other. Thus, the likelihood that a Dutch-English bilingual produces a code-switched sentence would be higher in “The boy puts the apple in the bag” than in “The boy puts the carrot in the bag,” because “apple” overlaps with its Dutch translation “appel” but “carrot” does not overlap with its Dutch translation “wortel”^2^. This triggering mechanism is in line with the ubiquitous finding that the activation of cognates in language processing leads to a relatively high level of cross-language activation in the bilingual’s mind, thus influencing language processing at both the lexical and sentence level (e.g., Costa et al., 2000; Van Hell and Dijkstra, 2002; Christoffels et al., 2007; Van Hell and De Groot, 2008; Van Assche et al., 2009, 2012; Soares et al., 2019). This cross-language activation caused by cognates makes both languages highly available for selection and can thus trigger the use of both languages in the same utterance.

Lexical triggering has mainly been studied by means of analyses of natural language corpora (Clyne, 1980; Broersma and De Bot, 2006; Broersma, 2009; Broersma et al., 2009, 2019), but some lab-based studies also examined lexical triggering in picture naming (Broersma, 2011) and sentence production (Kootstra et al., 2012; Bultena et al., 2015). For example, Broersma and De Bot (2006) counted code-switches in a corpus of conversations between Dutch/Moroccan-Arabic bilinguals and found that switches occurred more often in utterances containing a language-ambiguous word than in utterances without language-ambiguous words. Likewise, Broersma et al. (2019) performed an analysis of a large-scale corpus of conversations between Welsh-English bilinguals. They found not only that the presence of a cognate in an utterance facilitated this utterance to be code-switched, but also that code-switching was more frequent in bilinguals who produced relatively many cognates, that the likelihood of code-switching increased when there were more cognates in an utterance, and that a cognate in one clause can even influence the likelihood of code-switching in the same speaker’s next clause.

Two experimental studies examined lexical triggering in sentence production. Kootstra et al. (2012) investigated the role of, among other things, cognates in the primed production of code-switched utterances. Dutch-dominant Dutch-English bilinguals described target pictures by means of a code-switched sentence, after having been auditorily presented with a code-switched prime sentence. Kootstra et al. found that the tendency to code-switch at the same sentence position as in the prime sentence was indeed enhanced in trials in which one of the entities to describe was a cognate, yet only when the speakers were relatively proficient in English. Another study on lexical triggering in sentence production is Bultena et al. (2015). Bultena et al. (2015) investigated to what extent cognates would influence switch costs in sentence context. Rather than noun cognates, which are most often used in bilingual processing research, Bultena et al. (2015) focused on verb cognates. In their study, Bultena et al. applied a shadowing task, in which participants are auditorily presented with a sentence, which they have to repeat as directly and accurately as possible without waiting for the end of the recording; the latency between the onset of the original recording and the participant’s reproduction of it is regarded as a measure of processing time. They examined whether verb cognates would reduce shadowing latencies in code-switched sentences. Bultena et al. (2015) did find switch costs in the shadowing latencies (e.g., switching to L2 was more costly than switching to L1), but the switch costs were not modulated by the cognate manipulation, and this effect was not modulated by L2 proficiency.

Thus, whereas lexical triggering is observed in corpus research, the evidence on lexical triggering in sentence production experiments is a bit scarce and mixed. One factor that could play a role is the extent to which code-switched responses are internally generated versus externally induced (cf., Gullberg et al., 2009). In Kootstra et al. (2012) participants were indeed forced to produce code-switched sentences yet were free to choose how to code-switch. Thus, the responses in Kootstra et al. (2012) study could be regarded as internally generated, similar to corpus data. In contrast, Bultena et al. (2015) explicitly instructed participants to switch according to the sentence presented to them, which is less like corpus data. Another factor that may have played a role is that Kootstra et al. used noun cognates, whereas Bultena et al. focused on verb cognates. As made clear in another study by Bultena et al. (2013), effects of noun cognates tend to be stronger than those of verb cognates. Thus, it may well be that lexical triggering only emerges under specific circumstances and with specific types of words. An important goal of this study is to examine these possible circumstances and words in more detail.

With respect to which kinds of words may trigger code-switching, one question that has not yet been fully answered is how much cross-language overlap is needed for words to trigger code-switching. That is, in theory, not only cognates (sharing form and meaning across languages) could function as triggers but also *false friends* [sharing form but not meaning across languages, such as the Dutch-English ROCK-ROK (skirt)]. Like cognates, false friends have been found to induce co-activation in the bilingual mind (e.g., Dijkstra et al., 1999; Jared and Szucs, 2002; Schulpen et al., 2003; Haigh and Jared, 2008; Brenders et al., 2011; Carrasco-Ortiz et al., 2012), and they may therefore trigger code-switching. In a corpus analysis, Broersma et al. (2009) noted that code-switches sometimes co-occurred with false friends, but this observation has not yet been further tested. To investigate the psychological reality of this observation, we studied both false friends and cognates as potential triggers for code-switching. If false friends can indeed trigger a code-switch, this would imply that code-switching can be triggered by cross-language form overlap alone, independent from cross-language meaning overlap.

With respect to the circumstances under which lexical triggering may occur, a question that needs further scrutiny concerns the relative importance of lexical triggering compared with other forces on code-switching. Based on the concept of self-organized criticality, De Bot et al. (2009) argue that the lexical co-activation caused by trigger words is relatively small, and that triggering is probably most likely to occur in a setting where code-switching is already highly likely to occur. According to De Bot and colleagues, the tendency to code-switch is probably not caused by a single factor but by a constellation of different factors. When the conditions to code-switch are favorable, trigger words may provide the final nudge for bilinguals to switch to the other language. However, the mere occurrence of trigger words in a situation where code-switching is not likely to occur will not or hardly increase the likelihood of code-switching (as in Kootstra et al., 2012; Bultena et al., 2015). To systematically investigate this interpretation of triggered code-switching, it is important to compare lexical triggering of code-switching in conditions where code-switching is less likely to occur with conditions where code-switching is more likely to occur. This brings us to a second line of psycholinguistic research on bilinguals’ tendency to code-switch: interactive alignment.

Interactive alignment refers to the phenomenon that speakers in dialogue typically coordinate their language. They use the same words (Brennan and Clark, 1996), syntactic structures (Branigan et al., 2000), and even pronunciation (Pardo, 2006). Interlocutors thus activate the same linguistic representations and become *interactively aligned*. This copying of language use in discourse does not only foster communicative success (Pickering and Garrod, 2004; Garrod and Pickering, 2009; Menenti et al., 2012), but is also assumed to drive language learning and even language change (e.g., Ferreira and Bock, 2006; Garrod and Pickering, 2013; Kootstra and Şahin, 2018).

Although most research on interactive alignment in dialogue is based on monolingual speech, there is also evidence that interactive alignment takes place in bilingual dialogue. Treffers-Daller (1997) observed that a Turkish-German bilingual code-switched more when talking to a bilingual interlocutor than to a monolingual interlocutor. Likewise, Fokke et al. (2007) found that Dutch-English bilinguals code-switched more talking to a confederate who acted as a code-switching exchange student rather than as a non-code-switching monolingual student. These studies point to alignment of spontaneous code-switching, but the analyses are based on general discourse situations, and not on turn-by-turn alignment between listening and speaking. Kootstra et al. (2010), however, did observe turn-by-turn interactive alignment in code-switched dialogue. They asked pairs of Dutch-English bilinguals, one of which was a confederate, to code-switch while taking turns in describing pictures. Participants tended to copy the confederate’s word orders and code-switching patterns, which indicates that interactive alignment influences syntactic choice in code-switching.

Interactive alignment between utterances in bilingual dialogue was recently also investigated with respect to the actual choice to code-switch. Based on quantitative analyses of a large corpus of English–Spanish language use (the Bangor Miami Corpus; Deuchar et al., 2014), Fricke and Kootstra (2016) found that bilinguals’ tendency to code-switch in spontaneous bilingual dialogue was influenced by multiple factors relating to interactive alignment and priming. Importantly, the factor that influenced code-switching most systematically was the language of the preceding utterance. When the preceding utterance in the discourse was code-switched, the current utterance was also strongly likely to be code-switched. This effect took place both when the preceding utterance was produced by the dialogue partner (between-person priming) as well as by the same speaker (within-person priming). In addition, the effect was independent from effects of lexical overlap between the current and preceding utterance. That is, while lexical overlap between the current and preceding utterance influenced the tendency to code-switch in this corpus, the priming effect was still there after having factored out these effects of lexical overlap. Thus, it was not the case that findings of primed code-switching were based on lexical coherence between utterance (cf. Angermeyer, 2002). This evidence shows that, indeed, the tendency to code-switch is influenced by the presence of code-switches in the previous utterance.

How can we account for interactive alignment of code-switching from a psycholinguistic perspective? In monolingual dialogue, interactive alignment is accounted for by the *interactive alignment model* (Pickering and Garrod, 2004). This model specifies the processing levels (semantic, syntactic, lexical, phonological, and phonetic) that are involved in producing and comprehending linguistic messages in dialogue. The model’s basic principle is that representations that are activated to produce messages are also activated to comprehend messages; interactive alignment then occurs on the basis of residual activation of recently activated representations (i.e., priming; a mechanism that is not only relevant in between-person processes, but also influences language processing within persons; cf., Pickering and Ferreira, 2008). This creates a representational connection between interlocutors, leading to interactive alignment. To account for interactive alignment of code-switching, Kootstra et al. (2010) and Fricke and Kootstra (2016) extended the interactive alignment model with the assumption that lexical representations are connected to a language node, like in monologue models of bilingual language production (e.g., Kroll et al., 2006; Hartsuiker and Pickering, 2008; Kootstra et al., 2012). Interactive alignment of code-switching can then occur because listening to a code-switched utterance results in activation of language nodes from both languages, which increases the likelihood of subsequently producing an utterance with words from both languages (i.e., a code-switched utterance).

In short, bilinguals’ tendency to code-switch can be influenced by lexical triggering and by interactive alignment. The novel question we address in this paper is how lexical triggering *in combination* with interactive alignment influence speakers’ tendency to code-switch. More broadly, this will also shed light on the effects of and interactions between lexical and socio-interactional factors on bilingual speech in dialogue. We designed two dialogue experiments in which a confederate and a real participant took turns in describing a picture. The confederate’s switching was manipulated, and the participant could voluntarily code-switch or not in critical trials, in whatever direction of switching. This way, we created a situation in which variables were experimentally manipulated, but in which participants’ responses in critical trials were internally generated rather than externally induced, thus staying as close as possible to how natural code-switching in real-life dialogue takes place (see Gullberg et al., 2009, for further discussion on the importance of ecologically-valid lab-based approaches to code-switching at the sentence level, and see Gollan and Ferreira, 2009; Kleinman and Gollan, 2016; De Bruin et al., 2018; Jevtović et al., 2020, for similar points with respect to single-word language switching).

In Experiment 1, we investigated how lexical triggering in combination with interactive alignment affects the likelihood of producing code-switched sentences. We did this by analyzing Dutch-English bilinguals’ tendency to code-switch as a function of whether the confederate had code-switched in the previous turn (interactive alignment) and of whether the picture to be described contained a cognate, a false friend, or a control word (lexical triggering). If lexical triggering only occurs when conditions for code-switching are optimized (as argued by, e.g., De Bot et al., 2009), then lexical triggers are more likely to elicit a code-switch in a speaker when the previous speaker (i.e., the confederate) had just code-switched. Alternatively, if lexical triggering is a principled cognitive mechanism (i.e., language-ambiguous words like cognates or false friends co-activate two languages, which then elicits a switch to the other language), then lexical triggering would increase the likelihood of code-switching, irrespective of whether or not the confederate has just code-switched in the previous trial. Finally, if lexical triggering results not only from cognates but also from false friends, this would indicate that mere form overlap across languages, and not both form and meaning overlap, provides sufficient co-activation of languages to trigger a code-switch.

In Experiment 2, we zoomed in on the mechanism underlying interactive alignment of code-switching. That is, although interactive alignment of code-switching can elegantly be accounted for by combining the interactive alignment model with the notion of residual language activation of language nodes (as described above), interactive alignment could also be explained by assuming that bilinguals align on the act of code-switching. That is, as described in the pragmatic literature on code-switching (e.g., Tseng and Cashman, 2015), the act of code-switching in social interaction is considered to have conversational meaning in addition to an utterance’s semantic meaning. Thus, an utterance could be mentally represented as “switched” or “not-switched.” In theory, such an explanation does not necessitate the assumption of language nodes in the interactive alignment model. The language-activation account, however, is based on the residual activation of language node activation. This account does not only predict priming of code-switching after code-switched prime utterances, but also priming of code-switching by utterances in the “non-default” language, irrespective of whether the prime utterance is code-switched or not. For example, many Dutch-English bilinguals in the Netherlands are Dutch-dominant. This means that the default level of activation of Dutch will be higher than that of English. When such bilinguals are then exposed to a Dutch utterance, this will hardly change their relative activation of Dutch and English, because Dutch is already more strongly activated than English. If, however, these bilinguals are exposed to an English utterance, the level of activation of English will increase relatively strongly. This may lead to a situation where both Dutch (the dominant language) and English (the primed language) have a relatively high level of activation, thus enhancing the likelihood that both languages are used in the subsequent utterance. In Experiment 2, we contrasted the language-activation account with the pragmatic-act-of-code-switching account by testing Dutch-English bilinguals’ tendency to code-switch after an all-Dutch utterance, an all-English utterance, or a code-switched utterance. According to the language-activation account, the language activation from the previous utterance interacts with the level of language co-activation that was already present in the mind of the bilingual speaker who produces the next utterance, which entails that not only code-switched utterances but also all-English utterances could increase the likelihood of code-switching to occur. According the act-of-code-switching account, the tendency to code-switch should only be primed by code-switched utterances from the confederate; the language used in unilingual conditions should not have an influence on the participants’ likelihood to switch.

## Experiment 1

### Participants

Thirty-six students from Radboud University, Nijmegen, participated. All were Dutch native speakers who had started to learn English from 5th grade onward and were regularly exposed to English through popular media and study textbooks. Their scores on an English vocabulary test (L_Lex^3^; Meara et al., 2001) and their self-ratings revealed that they were relatively proficient yet Dutch-dominant speakers of English (see Table 1 for an overview of the participants’ characteristics). The participants reported that they code-switch regularly in their daily lives. The confederate’s language and educational background was comparable to the participants.

**TABLE 1:** Characteristics of participants in Experiment 1 and Experiment 2.

	Exp. 1 (*N* = 36)		Exp. 2 (*N* = 30)	
		
	*M*	*SD*	*M*	*SD*
Age	23.31	3.48	21.5	3.43
L_Lex vocabulary score^1^	76.08	11.76	77.03	10.32
Self-rated English skills^2^	5.51	0.71	6.06	0.54
Self-reported amount of CS^3^	2.98	0.82	2.94	1.36

*^1^L_Lex scores between 70 and 80 are equal to TOEFL (paper-based) scores 550–600. ^2^Seven-point scale: 1 = no ability; 7 = native-like ability. ^3^Five-point scale: 1 = never; 5 = always. CS refers to code-switching.*

### Materials

An experimental item was defined as a picture described by the confederate (prime) and a picture to be described by the participant (target). The prime-target picture pairs were line drawings of events (72 critical picture pairs and 36 filler picture pairs), involving an actor, action, patient, and prepositional phrase (in active sentence structure, e.g., “The hunter puts the rose on the chair”). See Table 2 for examples. The materials can be found in the online repository belonging to this study: https://doi.org/10.17026/dans-xyw-zp2u.

**TABLE 2:** Examples of critical trials in the Different Experimental Conditions of Experiment 1.

Word category	CS by confederate	Confederate’s prime utterance	Participant’s target picture
			
Cognate	Yes	De jager legt de *roos* on the chair.	grandma putting *baby* on chair
	No	De jager legt de *roos* op de stoel.	
		[The hunter puts the rose on the chair]	
False Friend	Yes	De duiker gooit de *rok*^1^ to the sailor.	waiter throwing *game*^2^ to sailor
	No	De duiker gooit de *rok*^1^ naar de matroos.	
		[The diver throws the box to the sailor]	
Control word	Yes	De slager neemt de *jas* from the wizard.	dentist taking *bike* from wizard
	No	De slager neemt de *jas* van de tovenaar.	
		[The butcher takes the coat from the wizard]	

*The words in italics are the critical words (the patients) that are manipulated in terms of cross-language overlap. The underlined words refer to the part of the confederate’s utterance that is switched. ^1^The Dutch word “rok” is the equivalent of English “skirt” and is a false friend with the English word “rock.” ^2^The Dutch word for “game” is “spel,” which is a false friend with the English word “spell.”*

To study lexical triggering, the patient in the prime-target picture pairs was a Dutch-English cognate (e.g., roos-rose; baby-baby), a false friend [e.g., rok (skirt)-rock; spel (game)-spell], or a control word (little to no cross-language phonological similarity, e.g., jas-coat; fiets-bike). To study interactive alignment, the confederate code-switched in half of the pictures and did not code-switch in the other half of the pictures. The confederate’s code-switch was always directly after the patient (a cognate, false friend, or control word to examine lexical triggering), from Dutch into English. The confederate always switched only once per utterance. The experimental manipulation led to six Confederate Code-switch (yes, no) × Trigger Word (cognate, false friend, control) conditions, see Table 2.

As can be seen in Table 2, there is overlap between the lexical materials used in the prime utterance and those in the target picture. First, lexical triggering (Word Category) was always manipulated in both the prime utterance and the target picture in such a way that the same word category was present in the prime and in the target. We did this to maximize the chance of finding a triggering effect, given that, so far, effects of lexical triggering in experimental sentence production tasks have proven to be rather elusive. Second, the confederate’s prime utterance and participant’s subsequent target picture always had the same action, the same actor or patient, and the same theme/location (i.e., the prepositional phrase). We did this to maximize the likelihood of interactive alignment to occur, given that previous research on priming and interactive alignment has shown that lexical overlap enhances priming effects in sentence production (e.g., Mahowald et al., 2016).

The fillers were also picture pairs, with different lexical items than the ones used in the critical trials. The filler target pictures were depicted on a red background, signifying that at least one English word had to be used in describing the picture. The background color in the fillers was added to make the confederate’s linguistic choices in the experiment, including the use of both Dutch and English in the critical trials, more natural, and create a situation in which it was normal to produce partly English (i.e., code-switched) utterances (cf., Kootstra et al., 2010). Importantly, the pictures in the critical trials were depicted on a white background, signifying that, in the critical trials, language choice was completely free and internally generated.

The 72 critical and 36 filler trials were randomized into six versions. All critical pictures were counterbalanced such that, across versions, each picture-pair occurred equally often in switch and non-switch conditions. Within each version, each individual word depicted in the pictures occurred equally often in each condition, and there were never two trials from the same condition in a row. To ensure that any effects would not be due to lexical items other than the critical words, all actors, actions, and prepositional phrases used to create the picture pairs occurred equally often in each condition.

### Procedure

To ensure that code-switching in the experimental task was not caused by word-finding difficulties, participants were first familiarized with the experimental materials by presenting the pictures with their Dutch and English names underneath, on a laptop (cf., Kootstra et al., 2010). The confederate and participant sat beside each other and took turns in naming the Dutch and English items.

After this, the actual experiment started. The confederate and participant sat opposite each other, both with a laptop in front of them. They were instructed to take turns in describing a picture and selecting the matching picture. Pictures had to be described in one complete sentence, using all elements that were depicted in the pictures (i.e., actor, action, patient, theme/location). Pictures with a white background (i.e., the experimental items) could be described in Dutch, English, or a combination of both, and there was no requirement to start these picture descriptions in a particular language. Thus, language choice in pictures with a white background was completely free. Pictures with a red background (i.e., the fillers) had to be described using at least one English word. Selecting the matching picture was done by pressing the key belonging to the described picture from two pictures on the screen. The confederate pretended to perform the same task as the real participant, but in fact read aloud the scripted picture description as it was typed out on the screen. The confederate always had the first turn.

Participants started with 12 practice trials and then completed the 108 experimental trials. Each participant was assigned one of the randomized versions described in the materials section. The experiment was run on E-Prime. All responses were recorded and later transcribed.

After the experimental task, the participants performed the L_Lex vocabulary task and completed a language history questionnaire that included self-ratings of their English proficiency and amount of code-switching in real life. The confederate was taken to another room to perform these additional tasks, but, in reality, simply left (and sometimes returned later for the next participant). The participant was told that the confederate was done sooner with the vocabulary tests if participants asked where the “other participant” went. The entire testing session lasted about 60 min.

### Scoring and Analysis

Each response was scored for whether it was code-switched (i.e., containing both Dutch and English words) or not. The data were then subjected to a mixed-effects logistic regression analysis, using the lme4-package (Bates et al., 2019) in R version 3.5.1 (R Core Team, 2018). With respect to our fixed effects, predictor variables were (1) Code-switching by the confederate (yes or no) and (2) Trigger word category (control word, cognate, false friend). The reference level for Code-switching by the confederate was “no,” and the reference level for Trigger word category was “control word.” Thus, effects of trigger word category are to be interpreted as the effect of trials with cognates vs. trials with control words and the effect of trials with false friends vs. trials with control words. The presence of an effect of trials with cognates vs. trials with control words would be evidence of cognate triggering, as found previously in corpus studies (Broersma and De Bot, 2006; Broersma, 2009; Broersma et al., 2009, 2019). An effect of trials with false friends vs. trials with control words would signify that even false friends can trigger code-switching to occur.

We started the analyses with a full model containing all predictors and interactions between the predictors, as well as random intercepts for participants and items, and by-participant random slopes for both Code-switching by the confederate and Trigger word category^4^. Subsequently, in a stepwise manner, we eliminated random slopes and tested the fit of the new model compared to the old model, using likelihood ratio tests. The reasoning behind this backward elimination is that if the fit of a simpler version of a model is not significantly different from the fit of a more complicated model, then the simpler model can be considered a more optimal reflection of the data (cf., Kootstra and Doedens, 2016). Effects of fixed-effects predictors were considered significant with *p*-values < 0.05.

### Results

The experiment yielded 2592 picture descriptions in critical trials, of which 135 (5.21%) were discarded because of an incomplete picture description. Of the remaining 2457 picture descriptions, 132 (5.37%) were discarded because a different word than the intended cognate or false friend was used. The analysis was based on the remaining 2325 responses (837 trials with control words, 790 trials with cognates, 698 trials with false friends).

**FIGURE 1:**
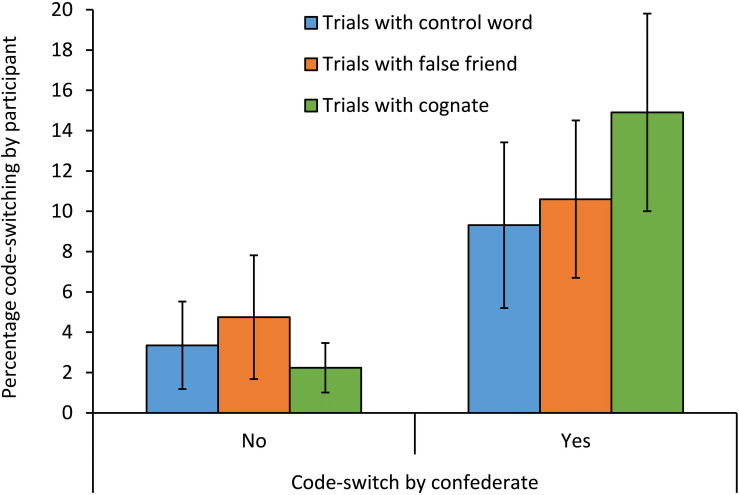
Percentages of code-switched responses per condition in the critical trials of Experiment 1. The error bars represent 95% confidence intervals.

The descriptive results are displayed in Table 3. A summary of the optimal mixed-effects analysis is given in Table 4. The optimal model of the mixed-effects analysis was the model containing only random intercepts for items and participants. This model yielded, firstly, a significant main effect of the Intercept. The negative value of the Intercept indicates that participants were significantly more likely not to code-switch than to code-switch. Secondly, the model yielded a significant main effect of Code-switching by the confederate. Participants switched significantly more often when the confederate had also switched than when the confederate had not switched. Thirdly, there was a significant interaction of Code-switching by the confederate with Trigger-word category, in the case of cognates compared to control words. As shown in Figure 1, the effect of Code-switching by the confederate was particularly strong in trials with cognates: Participants switched relatively frequently in pictures containing cognates when the confederate had just produced a code-switched utterance. There were no significant differences between trials with control words and trials with false friends, in main effects nor in interactions.

**TABLE 3:** Responses per condition in Experiment 1.

	No code-switch by confederate			Code-switch by confederate		
		
	Cognate	False friend	Control word	Cognate	False friend	Control word
N all-Dutch responses	377	331	386	307	286	334
N all-English responses	16	10	18	23	18	46
N code-switched responses	9	17	14	58	36	39

Percentage CS by participant	2.24	4.75	3.35	14.95	10.59	9.31

**TABLE 4:** Fixed effects of the optimal mixed-effects logistic regression model for variables predicting the likelihood of code-switching by the participant in Experiment 1.

	Estimate	*SE*	*z*-value	*p*-value
(Intercept)	–3.907	0.35	–11.149	<0.001
CSby confederate: yes (vs. no)	1.213	0.32	3.806	<0.001
Trigger word: cognate (vs. control word)	–0.448	0.47	–0.946	0.344
Trigger word: false friend (vs. control word)	0.389	0.42	0.931	0.352
CS by confederate x cognate (vs. control word)	0.981	0.48	2.034	0.042
CS by confederate x false friend (vs. control word)	–0.233	0.44	–0.529	0.597

### Discussion

The goal of Experiment 1 was to investigate how the combination of lexical triggering and interactive alignment influence bilinguals’ tendency to code-switch. In addition, we investigated to what extent not only cognates but also false friends could function as triggers for code-switching. The results indicated (1) that bilinguals were more likely to code-switch after the confederate had just switched than after the confederate had produced a unilingual utterance, (2) that trials with cognates indeed enhanced bilinguals’ tendency to code-switch, but only when the confederate had just code-switched, and (3) that trials with false friends did not result in a significantly increased tendency to code-switch.

The finding that bilinguals were more likely to code-switch after the confederate had just switched is evidence of interactive alignment of code-switching and shows that interactive alignment is an important predictor of code-switching. These findings parallel the findings on interactive alignment of code-switching in Fricke and Kootstra (2016). Importantly, whereas Fricke and Kootstra (2016) data were based on a corpus of spontaneous conversation, the current data were based on an experimental task. The fact that interactive alignment of code-switching has now been found in both an experimental and spontaneous setting adds to its robustness as a predictor of code-switching.

A second important result from Experiment 1 is that cognates indeed enhanced the chance of code-switching to occur, but only when the confederate had just switched. Thus, interactive alignment of code-switching appears to have functioned as a driving force for lexical triggering to occur. This is consistent with De Bot et al.’s (2009) idea that lexical triggering is only likely to effectuate a code-switch in a setting that is optimal for code-switching to occur. Interacting with someone who has just code-switched, as tested in Experiment 1, creates such a setting.

A third result from Experiment 1 is that trials with false friends did not lead to more code-switching than trials with control words. Thus, in the current experiment, false friends did not trigger code-switching. This indicates that form overlap alone is not a strong enough trigger for code-switching. This finding appears to be at odds with Broersma et al. (2009), whose corpus study did observe some code-switches preceded by false friends, as well as with previous studies showing evidence of co-activation of languages caused by false friends (e.g., Dijkstra et al., 1999; Schulpen et al., 2003; Haigh and Jared, 2008; Brenders et al., 2011; Carrasco-Ortiz et al., 2012). Apparently, when it comes to eliciting code-switches in sentences, cross-language overlap at both the phonological/orthographic and semantic levels, as in cognates, is important for lexical triggering to occur. We will elaborate on this in the General Discussion.

Thus, Experiment 1 provides evidence of both lexical triggering and interactive alignment of code-switching, showing that lexical triggering only occurs with cognates in a situational context where code-switching is already likely to occur (here: when the confederate had code-switched on the previous trial). The next question is which aspects of the confederate’s utterance the participants align with: the pragmatic act of code-switching (i.e., a mental representation at the pragmatic level of processing), or the actual language used by the confederate (i.e., mental representation of language node activation)? To investigate this question, we conducted an experiment in which the confederate not only produced all-Dutch or code-switched utterances, but also all-English utterances. According to the act-of-code-switching account, the tendency to code-switch should be influenced by whether the confederate code-switched or did not code-switch, irrespective of the specific language used in non-switched prime utterances. In contrast, according to the language-activation account, the language activation from the previous utterance interacts with the level of language co-activation that was already present in the mind of the bilingual speaker who produces the next utterance, which in Dutch-dominant Dutch-English bilinguals entails that not only code-switched utterances but also all-English utterances could increase the likelihood of code-switching to occur.

## Experiment 2

### Participants

Thirty new participants recruited from the same population as in Experiment 1 participated. The confederate’s language and educational background was comparable to the participants’ backgrounds. See Table 1 for an overview of the participants’ characteristics.

### Materials

As in Experiment 1, an experimental item was defined as a picture described by the confederate (prime) and a picture to be described by the participant (target). We created 40 critical trials and 60 filler trials. The critical trials were always ditransitive events or a transitive event with an object consisting of two themes. The materials can be found in the online repository belonging to this study: https://doi.org/10.17026/dans-xyw-zp2u.

To study interactive alignment of the act of code-switching versus language activation, we needed to come up with a design in which (1) the confederate either code-switches or does not code-switch and (2) the confederate either produces a Dutch-only utterance, an English-only utterance, or an utterance containing both English and Dutch. To fulfill this requirement, the confederate’s prime picture description was (1) an entirely Dutch utterance (10 items), (2) an entirely English utterance (10 items), or (3) a code-switched utterance (20 items; 10 with a switch from Dutch to English and 10 with a switch from English to Dutch). The critical trials thus consisted of equal numbers of switched and non-switched sentences. See Table 5 for examples.

**TABLE 5:** Examples of critical trials in Experiment 2, with conditions according to the language activation account and conditions according to the act-of-code-switching account.

Condition in terms of language activation account	Condition in terms of act-of-code-switching account	Confederate’s prime utterance	Participant’s target picture
Dutch	No CS	De slager zet de deksel op de vuilnisbak.	*Lumberjack throwing a band aid in a can*
English	No CS	The butcher puts the lid on the bin.	*Lumberjack throwing a band aid in a can*
Mixed	CS	The butcher puts the lid op de vuilnisbak./ De slager zet de deksel on the bin.	*Lumberjack throwing a band aid in a can*

*The underlined words refer to the part of the confederate’s utterance that is switched. Just as in Experiment 1, the confederate’s code-switch was always directly after the patient.*

A difference between the stimuli in Experiment 2 compared to Experiment 1 is that there was no lexical overlap between the primes and targets in the critical trials. We did this to ensure that any effects of interactive alignment could only be accounted for by alignment of the act of code-switching or alignment of language choice, and not by other factors known to influence interactive alignment, such as lexical coherence between the prime and target (cf., Angermeyer, 2002; Fricke and Kootstra, 2016). In addition, because this experiment focused purely on alignment effects and not on lexical triggering, we made sure that no cognates or false friends were used in the critical trials.

The fillers were also picture pairs, but none of the lexical items appeared in the critical trials. The filler target pictures were depicted on a red or a blue background. The red background signified that at least one Dutch word had to be used in describing the picture, while the blue background signified that at least one English word had to be used in describing the picture. Background colors were added in the filler trials to make the confederate’s linguistic choices in the experiment more natural, and to create a situation in which the use of Dutch, English, and switching in the critical trials is not unexpected. As in Experiment 1, critical-trial target pictures were depicted on a white background, signifying that language choice was completely free.

The critical and filler trials were distributed across four lists, in which each prime-target combination occurred only once and was rotated across conditions between each list. Within each list, each individual word depicted in the pictures occurred equally often in each condition. To ensure that any effects would not be due to lexical items other than the critical words, all actors, actions, and prepositional phrases used to create the picture pairs occurred equally often in each condition. Each list was randomized into three versions, in which we made sure that code-switch and non-code-switch trials were unpredictably and evenly distributed across the list.

### Procedure

The procedure was the same as in Experiment 1, except that there were not only pictures with a red background or a white background, but also pictures with a blue background. The participants were instructed to use at least one Dutch word when the picture’s background was red, and at least one English word when the picture’s background was blue. In pictures with a white background (i.e., the experimental items), participants were free to describe them in Dutch, English, or a combination of both languages, just as in Experiment 1.

### Scoring and Analysis

As in Experiment 1, each response was scored for whether it was switched or not. We then built two separate mixed-effects models on these data, using the same procedure as in Experiment 1. In the first model, the fixed-effects predictor is “Code-switching by the confederate” (yes or no, where “yes” refers to confederate’s utterances that were code-switched, and “no” refers to all confederate’s utterances that were either all-Dutch or all-English). In the second model, the fixed-effects predictor is “Language used by the confederate” (“Dutch,” “English,” “Mixed”). The first model can be seen as a replication of the confederate’s code-switching effect from Experiment 1, and taps into the question whether the act of code-switching by the confederate, irrespective of the language used by the confederate in no-switch conditions, influences the participants’ tendency to code-switch. The second model is an elaboration of the first model and addresses the question to what extent the specific language used by the confederate influences the participants’ tendency to code-switch. To assess which of these models provides the best explanation of the data, the fit of both models will be compared using likelihood ratio tests. If the fit of Model 1 and Model 2 are not significantly different from each other, then the act of code-switching by the confederate, irrespective of language used in the no-switch conditions, can be seen as the most optimal explanation of interactive alignment in code-switching (if this predictor reaches significance at all, of course). After all, Model 1 is a simpler model (in terms of degrees of freedom) than Model 2. If, however, the fit of Model 2 (which is more elaborate than Model 1) is significantly better than the fit of Model 1, then “Language used by the confederate” is a better predictor of participants’ code-switching behavior. In the latter case, this should then of course also be reflected in significant effects of Language used by the confederate.

### Results

The experiment yielded 1200 picture descriptions in critical trials, of which 29 (2.42%) were discarded because of an incomplete picture description. The analysis was based on the remaining 1171 responses.

The descriptive results are presented in Table 6. A summary of the optimal mixed-effects analysis using “Code-switching by the confederate” as its predictor (Model 1) is given in Table 7; a summary of the optimal mixed-effects analysis using “Language used by the confederate” as its predictor (Model 2) is given in Table 8. In both cases, the optimal model was the model containing only random intercepts for items and participants. First, both Models 1 and 2 yielded a significant main effect of the Intercept. As in Experiment 1, the negative value of the Intercept indicates that participants were significantly more likely not to code-switch than to code-switch. In addition, Model 1 yielded a significant main effect of Code-switching by the confederate. Like in Experiment 1, participants switched significantly more often when the confederate had also switched than when the confederate had not switched. The pattern of results in Model 2, however, shows that the actual language use by the confederate also matters. The significant effect of Dutch vs. code-switched utterances indicates that participants’ tendency to code-switch after the confederate had just code-switched is significantly stronger than when the confederate had just produced a Dutch-only utterance. The non-significant effect of English vs. code-switched utterances indicates that participants’ tendency to code-switch after the confederate had just code-switched is *not* significantly different from when the confederate had produced an English-only utterance. Importantly, when comparing the fit of Model 1 with Model 2, it appeared that Model 2 had a much better fit than Model 1 (Model 1: log likelihood = −5864.5, *df* = 5; Model 2: log likelihood = −5853.8, *df* = 6; likelihood ratio test: χ^2^(1) = 21.488, *p* < 0.001). This indicates that “Language used by the confederate” provides a better explanation of the data than “Code-switching by the confederate.”

**TABLE 6:** Responses per condition in Experiment 2.

	Confederate’s prime utterance		
	
	Mixed	English	Dutch
N all-Dutch responses	229	109	151
N all-English responses	223	128	95
N code-switched responses	131	58	47

Percentage code-switching by participant	22.47	19.66	16.04

**TABLE 7:** Fixed effects of the optimal mixed-effects logistic regression model based on code-switching by the confederate, Experiment 2.

	Estimate	*SE*	*z*-value	*p*-value
(Intercept)	–2.0253	0.2709	–7.334	<0.001
CSby confederate: yes (vs. no)	0.3661	0.162	2.259	0.024

**TABLE 8:** Fixed effects of the optimal mixed-effects logistic regression model based on language used by the confederate, Experiment 2.

	Estimate	*SE*	*z*-value	*p*-value
(Intercept)	−1.6606	0.2713	−6.122	<0.001
Confederate’s language is English (vs. CS)	−0.216	0.1959	−1.103	0.270
Confederate’s language is Dutch (vs. CS)	−0.5283	0.2063	−2.561	0.010

A reviewer pointed out that the data may also be informative on scenarios of inter-sentential switching in dialogue, as reflected in the likelihood that participants begin their utterance with the language last used by the confederate. Analyses that explore this suggestion are reported in the Supplementary Material. In short, these analyses confirm that participants adjust their linguistic choices, including their code-switching tendencies, to the confederate’s patterns of language use, but they do not show the specific tendency to begin their response with the language last used by the confederate.

### Discussion

The goal of Experiment 2 was to elucidate at which level of processing interactive alignment takes place: at the level of the pragmatic act of code-switching or at the level of language node activation?

The results from Experiment 2 indicate that the model based on language use by the confederate (language activation account) has a better fit with the data than the model based on whether the confederate had just code-switched or not (act-of-code-switching account). Thus, the language activation account provides a more complete explanation of our data than the act-of-code-switching account, consistent with previous accounts of language co-activation as an explanation for code-switching.

The language node activation account is consistent with previous accounts of interactive alignment in code-switching (Kootstra et al., 2010, 2012; Fricke and Kootstra, 2016). What is more, Fricke and Kootstra (2016), who studied Spanish-English code-switching on the basis of a corpus of naturalistic speech, in fact observed similar patterns of code-switching after non-default language primes as in the current study (the default language was specified per conversation in the corpus Fricke and Kootstra analyzed, and could either be Spanish or English; it was defined as the language that was used most in the conversation). That is, in addition to observing robust evidence of code-switching when the previous utterance was code-switched, Fricke and Kootstra (2016) also found that, in conversations that were mainly in English (default-English conversations), the likelihood of code-switching increased when the previous utterance was all-Spanish. Although this pattern of results was less clear in default-Spanish conversations, it does suggest that unilingual utterances from the non-default languages can lead to primed code-switching. The results from the current study provide further empirical support for this account.

An additional observation from Experiment 2 is that the interactive alignment effects were found based on stimuli in which there was no lexical overlap between the prime utterances and target pictures. Lexical overlap between utterances in bilingual discourse has been found to increase the likelihood of code-switching to occur and can thus serve as an explanation of how code-switching in one utterance can be influenced by specific words used in a specific language in previous utterances (Angermeyer, 2002; Fricke and Kootstra, 2016). The fact that we found evidence of interactive alignment in the absence of lexical overlap between primes and targets indicates that, although lexical coherence between primes and targets affects the likelihood of code-switching, it is not necessary for interactive alignment of code-switching to occur. This corroborates corpus-based findings by Fricke and Kootstra (2016), who also found that priming of code-switching can take place in the absence of lexical coherence between prime and target. Our findings thus substantiate the idea that interactive alignment of language choice in bilingual discourse can take place at the “abstract” level of language nodes: Lexical coherence is not necessary for interactive alignment to occur.

## General Discussion

In two confederate-scripted dialogue experiments, we investigated to what extent the combination of lexical triggering and interactive alignment affect the likelihood of Dutch-English bilinguals to code-switch, and how effects of interactive alignment on code-switching can be accounted for. The results indicate that Dutch-English bilinguals have a stronger tendency to code-switch in trials containing cognates compared to non-cognates, but only when the confederate had just code-switched in the previous trial. However, they did not have a stronger tendency to code-switch in trials with false friends. The results of Experiment 2 provide further evidence that Dutch-English bilinguals align their language choices with their dialogue partner, and that this behavior is best explained by alignment of language activation rather than alignment of the act of code-switching.

The finding that trials with a cognate were code-switched more often than trials with a control word provides support for the lexical triggering hypothesis, and substantiates the evidence on lexical triggering found so far (Broersma and De Bot, 2006; Broersma, 2009; Broersma et al., 2009, 2019). An important difference between the present study and previous studies reporting evidence that supports the lexical triggering hypothesis is that the current study is based on experimental data, whereas previous studies were principally based on corpus data. Although corpora are, of course, optimal in terms of ecological validity, empirical studies are optimal for explicitly manipulating variables in combination with other predictors likely to influence code-switching. Using an experimental design, lexical triggers (i.e., cognates but not false friends) did indeed elicit code-switched utterances in speakers, but only when code-switching was already likely to occur, namely when their interlocutor had just code-switched. This conclusion is consistent with De Bot et al.’s (2009) notion that lexical triggering as a mechanism for code-switching is particularly likely to occur when the conditions for code-switching are already favorable. Importantly, we were able to draw this conclusion because of our experimental manipulation of lexical triggering in combination with another predictor of code-switching, interactive alignment. Corpus analysis alone would make it more difficult to specify discourse-related conditions that constrain lexical triggering. This also speaks to the importance of investigating code-switching using multiple approaches (see Gullberg et al., 2009; Van Hell et al., 2015, 2018; Beatty-Martínez et al., 2018; Munarriz-Ibarrola et al., 2018; Stadthagen-González et al., 2018; Valdés Kroff et al., 2018).

Our second finding on lexical triggering was that trials with false friends did not result in more code-switching than trials with control words. This finding provides important insights into the locus of the cognate triggering effect: It indicates that cross-language phonological overlap alone does not affect the likelihood of code-switching, but that semantic overlap is needed as well. The most plausible explanation of lexical triggering then is that lexical triggering is effectuated by the co-activation of translation equivalents (comprising phonological and semantic levels), rather than merely the co-activation of phonologically similar words that do not share semantics (and are thus not translation equivalents). This explanation is in line with related work on bilingual language production, stating that bilingual word production inherently entails a co-activation of its translation equivalent via the shared conceptual node (see, e.g., Costa et al., 2000; Forster and Jiang, 2001; Kroll et al., 2006; Dimitropoulou et al., 2011; Kootstra and Doedens, 2016; Broersma et al., 2019).

The interactive alignment findings in the current study not only show that interactive alignment is an important predictor of code-switching behavior, but also provide further insight into the underlying mechanisms. As the results from Experiment 2 suggest, interactive alignment of code-switching can best be explained by means of the alignment of residual language node activation. More specifically, when a dialogue partner uses Language A, Language B, or a combination of Languages A and B, this will lead to the activation of these languages via language nodes that are connected to lexical representations in the mental lexicon (cf., Hartsuiker and Pickering, 2008; Kootstra et al., 2012). Residual activation of this language activation pattern can then influence language choice in the subsequent utterance. Importantly, this language activation account assumes that residual activation from the previous utterance interacts with the level of language co-activation in bilingual speaker who produces the next utterance. In this case, even unilingual utterances can shift the level of language co-activation in the bilingual speaker who is about to produce the next utterance. This account of interactive alignment of code-switching in terms of alignment of language activation is consistent with Kootstra et al.’s (2010) and Fricke and Kootstra’s (2016) findings. It extends the interactive alignment model with the assumption that lexical representations are connected to language nodes, like in monologue models of bilingual language production (e.g., Kroll et al., 2006; Hartsuiker and Pickering, 2008; Kootstra et al., 2012).

Our interpretation of interactive alignment of language activation parallels a recent study by Pérez et al. (2019). Using the technique of electroencephalographic hyperscanning, Pérez et al. (2019) found that similarities in brain activation patterns between speakers and listeners in conversation depended on the language used in the conversation. As Pérez et al. (2019) argue, these similarities in brain activation patterns can be regarded as a neural approximation of the representational connection between interlocutors, in line with Pickering and Garrod’s interactive alignment model. This indicates that interlocutors align on language choice.

It is relevant to note that our experiments sought to combine experimental rigor with ecological validity. Models of bilingual language production are mostly based on reaction time experiments in monologue or on syntactic choices in dialogue in which language choice was imposed on participants (but see Gollan and Ferreira, 2009; Kleinman and Gollan, 2016; De Bruin et al., 2018; Jevtović et al., 2020, for single-item switching monologue tasks based on free language choice). The present study demonstrates that the mechanism of co-activation as specified in models of bilingual language production extends to interactive alignment in bilingual dialogue when language choice is completely free, which is a close approximation of natural language use. Moreover, the methodology of testing spontaneous code-switching in dialogue with experimental control provides a bridge between corpus studies on spontaneous code-switching in natural discourse (e.g., Fricke and Kootstra, 2016; Broersma et al., 2019), laboratory studies on lexical processing of cognates (e.g., Costa et al., 2000; Van Hell and Dijkstra, 2002; Christoffels et al., 2007; Van Assche et al., 2009), and studies on interactive alignment and structural priming (e.g., Pickering and Garrod, 2004; Hartsuiker and Pickering, 2008; Kootstra et al., 2010, 2012; Van Gompel and Arai, 2018).

There are at least two avenues for future research. Firstly, in Experiment 1, the trigger word manipulation occurred in both the primes and the targets. We did this to maximize the chance to find any triggering effects, but this does not make it possible to disentangle potential effects of triggering in the prime utterances from effects of triggering in the target picture descriptions. An idea for future research would be to manipulate the presence of trigger words in the primes and targets independent from each other. This would not only provide more insight into sources of triggering in code-switching (cf., De Bot et al., 2009; Broersma et al., 2019), but also into how processes of language comprehension and language production are related to each other (see e.g., MacDonald, 2013; Pickering and Garrod, 2013; Dell and Chang, 2014; Guzzardo Tamargo et al., 2016; Litcofsky and Van Hell, 2019). A second line of future research would be to further explore to what extent variation in bilinguals’ background variables, such as their relative proficiency in both languages or the frequency and contexts in which they code-switch in their daily lives, influences variation in code-switching behavior. As argued by multiple researchers, individual variation in language experience and proficiency shapes linguistic behavior, and provides an important methodological tool to test theories on how such language experiences influence language processing and language learning (e.g., Green and Abutalebi, 2013; Beatty-Martínez and Dussias, 2017; Kidd et al., 2018; Fricke et al., 2019). The participants in the current study were all from the same population and differed little in terms of proficiency and linguistic experiences. Not surprisingly, therefore, these background variables did not influence the results in the current study. To further investigate the potential role of individual differences in such background variables, larger and more varied groups of bilinguals should be studied. Although this is by no means easy to organize, it provides an important path to gain more insight into the complexity and adaptivity of linguistic behavior in multilingual settings.

To conclude, this study provides empirical evidence of an interplay between socio-interactional and lexical processes in code-switching in dialogue. Both these processes can be explained by resorting to the notion of co-activation of languages, which plays a central role in many theories on bilingual processes. Our experiments clarify how co-activation of languages at multiple levels of processing influences the bilinguals’ tendency to code-switch. In all, our findings call for a perspective on bilingual language production in which cross-speaker and cross-language processes are combined.

## Data Availability Statement

The datasets analyzed for this study can be found here: https://doi.org/10.17026/dans-xyw-zp2u.

## Ethics Statement

Ethical review and approval was not required for the study on human participants in accordance with the local legislation and institutional requirements. The patients/participants provided their written informed consent to participate in this study.

## Author Contributions

GK: study design, preparing and programming the experiments, data collection, data analysis and interpretation, and preparing and revising the manuscript. TD and JH: study design, data interpretation, and revising the manuscript. All authors contributed to the article and approved the submitted version.

## Conflict of Interest

The authors declare that the research was conducted in the absence of any commercial or financial relationships that could be construed as a potential conflict of interest.

## Footnotes

1 In the original version of the triggering hypothesis as proposed by Clyne (1980), trigger words include loanwords, bilingual homophones (including cognates), proper nouns, and compromise words (i.e., words that constitute a compromise form between two languages). The more recent empirical literature, however, has operationalized trigger words mostly as cognates (and sometimes as false friends; see e.g., Broersma et al., 2009; this article).

2 Note that the mechanism of lexical triggering is about how characteristics of a trigger word in a sentence influence the likelihood that *other* words downstream that sentence will be code-switched. It is not about how characteristics of a word (e.g., accessibility or frequency) influence the likelihood of that specific word to be switched. Such lexical influences on code-switching are typically investigated in studies on (voluntary) switching in single-word picture naming (e.g., Gollan and Ferreira, 2009; Kleinman and Gollan, 2016; De Bruin et al., 2018).

3 The L_Lex test is a lexical decision task of English that was developed by Lognostics (http://www.lognostics.co.uk). The L_Lex test is not available online anymore, but it is highly similar to Lognostics’ V_YesNo task, which can be found here: http://www.lognostics.co.uk/tools/.

4 By-item random slopes were not included, because the items were defined by trigger word category; in other words, there was no within-item variation across conditions.
