# Supplementary material for: Interactive Alignment and Lexical Triggering of Code-Switching in Bilingual Dialogue
Source: Front Psychol. 2020 Jul 22;11:1747. doi: 10.3389/fpsyg.2020.01747 (PMC7387648; doi:10.3389/fpsyg.2020.01747)
Supplement: Supplementary file 1 [file Data_Sheet_1.PDF]

Supplementary Material

Supplementary Analysis to explore scenarios of inter-sentential switching

A reviewer pointed out that the data may also be informative on scenarios of inter-sentential switching in dialogue, as reflected in the likelihood that participants begin their utterance with the language last used by the confederate. This provides further insight into interactive alignment in dialogue and how interlocutors accommodate their language choices. We therefore explored whether there was a relationship between the language with which participants began their responses and the confederate's prior utterance. We did this only for the data in Experiment 2, because in this experiment half of the confederate's utterances were switched from Dutch to English and the other half were switched from English to Dutch. Thus, any potential effects of the confederate's switching direction could be taken into account. We could not do this for the data in Experiment 1, because in this experiment the confederate always switched from Dutch to English.

Table 1 (see next page) presents the participants' responses per condition as a function of the confederate's prior utterance. One pattern that emerges is that participants' code-switched responses were more often switched from English to Dutch than vice versa, but the preference for switching from English to Dutch is less pronounced when the confederate had produced an all-Dutch utterance on the previous trial. A mixed-effects logistic regression analysis confirms this pattern of results: the tendency to produce a code-switch from English to Dutch vs. a code-switch from Dutch to English was significantly different when the prime utterance was all-Dutch compared to when the prime utterance was all-English ($\beta = 0.997$, $SE = 0.487$, $z = 2.05$) or code-switched from Dutch to English ($\beta = 1.114$, $SE = 0.501$, $z = 2.22$), and marginally significantly different compared to when the prime utterance was code-switched from English to Dutch ($\beta = 0.863$, $SE = 0.468$, $z = 1.84$). What this pattern of results indicates is that participants indeed appear to adjust their code-switching patterns to the language used in the utterance they were primed with. It does not follow from the data, however, that participants had the tendency to begin their response with the language last used by the confederate. If this had been the case, there should have been more responses with a code-switch from Dutch to English when the prime utterance was code-switched from English to Dutch.

A second pattern of results from Table 1 is that when participants produced a non-switched response after the confederate had switched on the previous trial, participants more often produced an all-Dutch utterance when the confederate's prime utterance contained a code-switch from Dutch to English, whereas they more often produced an all-English response when the prime utterance contained a code-switch from English to Dutch. This pattern of results is also confirmed by a mixed-effects logistic regression analysis: the tendency to produce an all-Dutch vs. an all-English response

after a code-switched prime utterance was significantly different between when the prime utterance was code-switched from Dutch to English compared to when it was code-switched from English to Dutch (β = -0.575, *SE* = 0.264, *z* = -2.179). While this result again indicates that participants adjust their linguistic choices to the confederate's patterns of language use, it also shows that participants did not have the tendency to begin their response with the language last used by the confederate. Rather, it seems that the participants tended to use the language first used in the confederate's prime utterance.

Table 1. *Participants' responses per condition in Experiment 2 as a function of the confederate's prior utterance*

	Confederate's prime utterance			
	all-Dutch	all-English	CS English to Dutch	CS Dutch to English
N all-Dutch responses	151	109	105	124
N all-English responses	95	128	116	107
N CS responses from English to Dutch	27	43	52	45
N CS responses from Dutch to English	20	15	19	15

Note. 'CS' refers to 'code-switched'.
